# Mechanistic signs of double-barreled structure in a fluoride ion channel

**DOI:** 10.7554/eLife.18767

**Published:** 2016-07-23

**Authors:** Nicholas B Last, Ludmila Kolmakova-Partensky, Tania Shane, Christopher Miller

**Affiliations:** Department of Biochemistry, Howard Hughes Medical Institute, Brandeis University, Waltham, United States; National Institutes of Health, United States

**Keywords:** fluoride ion channel, permeation, membrane protein architecture, *E. coli*, Other

## Abstract

The Fluc family of F^−^ ion channels protects prokaryotes and lower eukaryotes from the toxicity of environmental F^−^. In bacteria, these channels are built as dual-topology dimers whereby the two subunits assemble in antiparallel transmembrane orientation. Recent crystal structures suggested that Fluc channels contain two separate ion-conduction pathways, each with two F^−^ binding sites, but no functional correlates of this unusual architecture have been reported. Experiments here fill this gap by examining the consequences of mutating two conserved F^−^-coordinating phenylalanine residues. Substitution of each phenylalanine specifically extinguishes its associated F^−^ binding site in crystal structures and concomitantly inhibits F^−^ permeation. Functional analysis of concatemeric channels, which permit mutagenic manipulation of individual pores, show that each pore can be separately inactivated without blocking F^−^ conduction through its symmetry-related twin. The results strongly support dual-pathway architecture of Fluc channels.

**DOI:** http://dx.doi.org/10.7554/eLife.18767.001

## Introduction

**Figure 1.**
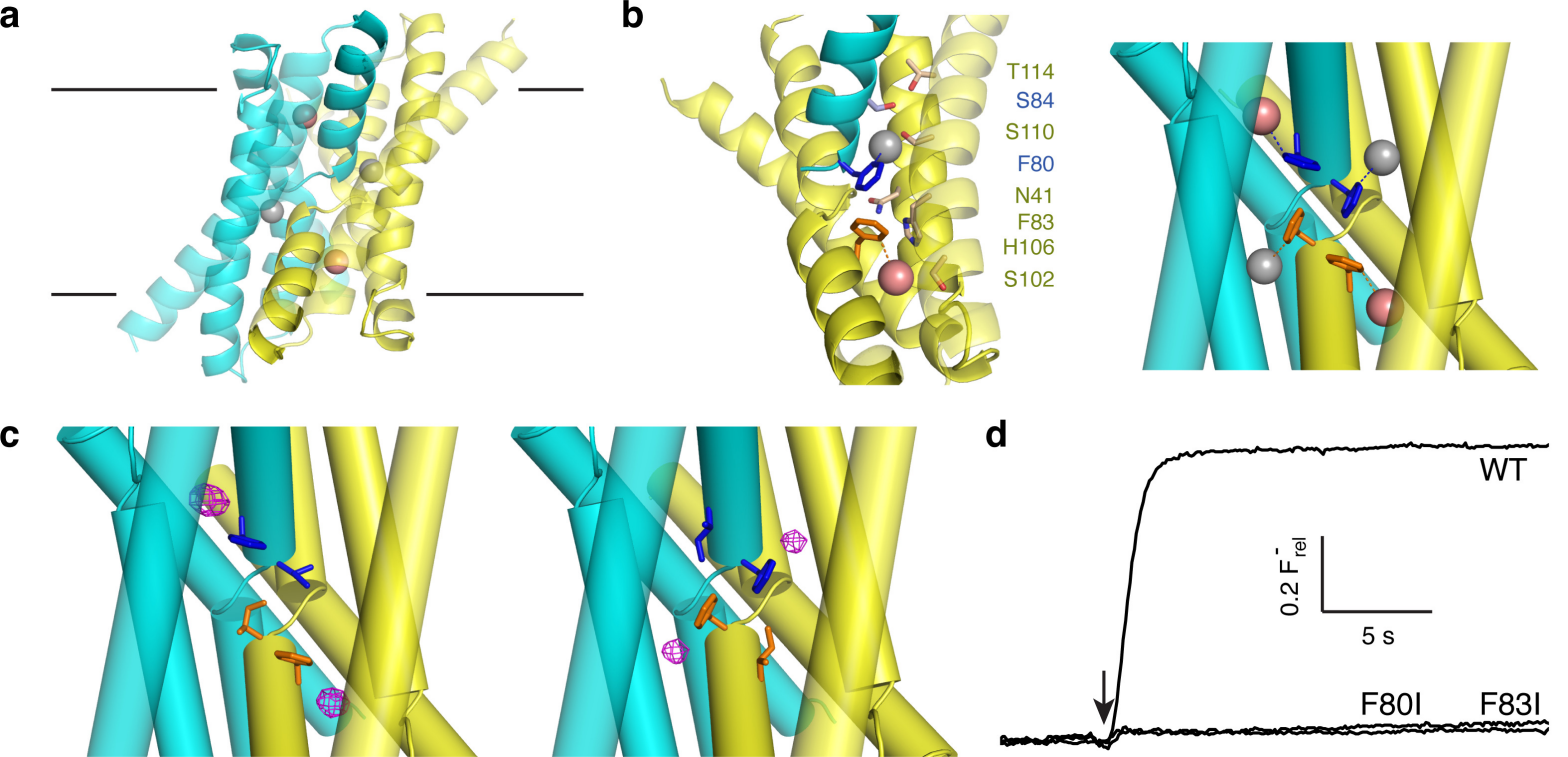
Fluoride ions in Fluc-Ec2 channels. (**a**) View of Ec2 channel imagined in membrane (black lines) with subunits colored cyan and yellow and F_1_^-^, F_2_^-^ ions indicated by grey, pink spheres, respectively. From PDB #5A43, rerefined to 2.56 Å resolution. (**b**) Ion-coordinating region of WT Ec2. 'Polar track' along one pore (left), and detail of the F^−^-binding Phe-box region (right). The polar track and Phe-box contain residues from both dimer subunits, with sidechains colored accordingly. (**c**) Phe-box region of F80I (left) or F83I (right) crystal structures (PDB 5KBN, 5KOM, respectively). F^−^-omit difference densities are shown in magenta, contoured at 4 σ. (**d**) F^−^ flux traces of indicated Fluc variants. Vertical scale bar represents F^−^ appearing in external medium after addition of valinomycin (arrow). **DOI:** http://dx.doi.org/10.7554/eLife.18767.002

The F^−^ ion, ubiquitous in the aqueous biosphere since the dawn of life, has been generally considered an irrelevant nonparticipant in membrane biology. This view was recently upended by the discovery of F^−^-specific riboswitches in many bacterial genomes (Baker et al., 2012), a breakthrough that over the past few years has revealed F^−^-transporting membrane proteins underpinning a widespread microbial physiology of resistance to environmental F^−^ toxicity (Baker et al., 2012; Stockbridge et al., 2012; Li S et al., 2013; Ji et al., 2014; Smith et al., 2015). These F^−^ exporters fall into two phylogenetically unrelated classes: energy-consuming anion transporters of the CLC superfamily (Stockbridge et al., 2012; Brammer et al., 2014), and electrodiffusive, F^−^-specific ion channels of the Fluc family (Stockbridge et al., 2013). While CLC proteins have been studied for decades (Miller, 2015), Flucs represent a novel and idiosyncratic class of ion channels. Bacterial Fluc channels assemble as dimers of small subunits (120–130 residues, 4 transmembrane helices) arranged in antiparallel transmembrane topology. This dual-topology architecture, originally inferred from biochemical and electrophysiological behavior (Stockbridge et al., 2013; Stockbridge et al., 2014), was recently observed in crystal structures of two homodimeric Fluc homologues (Stockbridge et al., 2015), as depicted in Figure 1a. The hourglass-shaped channel is complexed with two 'monobody' crystallization chaperones, which plug each of the wide vestibules facing the two aqueous solutions. (Figure 1—figure supplement 1). These ~10 kDa engineered monobodies are also high-affinity channel blockers whose long-lived block-times are readily observed in single-channel recordings (Stockbridge et al., 2014).

**Figure 1—figure supplement 1.**
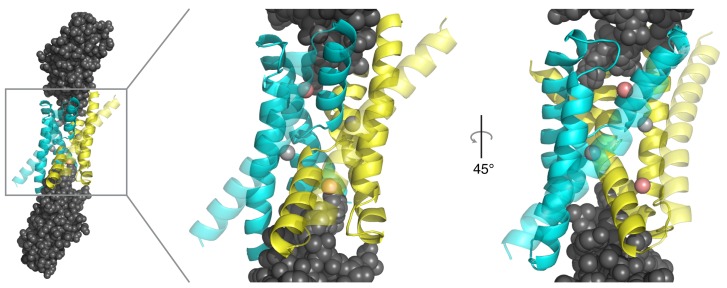
Monobody insertion into Fluc aqueous vestibules. Structure of WT Ec2 channel (PDB #5A43), also depicting S9 monobodies (grey) bound to each vestibule. **DOI:** http://dx.doi.org/10.7554/eLife.18767.003

The structures suggested an additional surprising feature of Fluc proteins: double-barreled architecture. In contrast to most ion channels, in which a single ion-permeation pore spans the membrane along a central axis, Flucs were proposed to contain two pores running along the sides of the complex, with each pore built by residues from both subunits (Stockbridge et al., 2015). However, this is a crystallographically 'dry' inference based on structural results alone, unaccompanied by any functional behavior that would add mechanistic vitality to support or refute this otherwise unprecedented picture. An additional crystallographic ambiguity is that the crevices housing the anion densities are too narrow to show themselves at 2.1–2.6 Å resolution as clearly delineated aqueous pores traversing the protein. For these reasons, we consider that the argument for dual-pathway construction is not yet consummated; it calls for tests based on functional properties of the pores manipulated individually. By solving crystal structures of ion-permeation mutants of a bacterial Fluc channel and analyzing the behavior of fused-dimer constructs, we now unambiguously establish the two-pore character of Fluc channels.

## Results

All experiments here employ the previously described 'Ec2' Fluc homologue from an *E. coli* virulence plasmid (Stockbridge et al., 2013). The homodimeric channel's symmetric, dual-topology structure demands that the pores adopt antiparallel orientations and that the two chemically distinct F^−^ ions in one pore, designated F_1_^−^ and F_2_^−^, are mirrored symmetrically in the other. In each pore, the two ions are coordinated by dipolar H-bond donors situated along a transmembrane 'polar track' (Figure 1b) that constitutes a possible ion pathway through the channel. Moreover, the F^−^ coordination shells are completed by conserved phenylalanine residues interacting edge-on with the bound anions (Figure 1b), Phe80 associated with the F_1_^−^ pair and Phe83 with the F_2_^−^ pair. These four aromatic side chains adopt a striking side-to-face 'Phe-box' arrangement that supports an unusual aromatic-halide coordination motif proposed to be essential for F^−^-specific binding and permeation (Stockbridge et al., 2015). Edge-on aromatic-anion coordination geometry makes chemical sense in light of the electropositive hydrogens of the quadrupolar aromatic ring (Jackson et al., 2007; Philip et al., 2011; Schwans et al., 2013). The two antiparallel polar tracks and their associated bound F^−^ ions are well-separated in the crystal structure, with no plausible pathway for ions to transfer between tracks. These structural features led to the proposal, to be tested here, that the two polar tracks represent two separate pores.

### Phe-box residues are essential for F^−^ binding and transport

Mutation of the conserved Phe-box residues in a Fluc homologue from *B. pertussis* was previously shown to severely inhibit F^−^ transport, an effect presumed to reflect the replacement of F^−^-friendly binding sites in the conduction pathways with kinetic barriers to ion movement (Stockbridge et al., 2015). This idea is now tested in Ec2, where we here examine occupancy of the F^−^-binding sites by solving crystal structures of Phe-box mutants F80I and F83I (Figure 1c, Table 1) and assessing their transport function. Substitution of each aromatic side chain by Ile specifically extinguishes the corresponding pair of F^−^ densities, one in each pore, while leaving the other pair Phe-coordinated and unperturbed. The mutations cause only local structural disturbances such as alternate rotamers at nearby sidechains Ser84 and Ser110 (Cα rmsd 0.4 Å, 0.2 Å for F80I, F83I). Functionally, the mutations are dramatic, reducing F^−^ flux in reconstituted liposomes to undetectable levels (Figure 1d), a >10^4^-fold rate inhibition compared to the wildtype (WT) channel (Stockbridge et al., 2013). These facts imply that Phe80 and Phe83 lie along F^−^ permeation pathways and that the functional defect in the mutants stems directly from the loss of the aromatic ring as a F^−^-binding partner in these pathways.

**Table 1.** Data collection and refinement statistics. **DOI:** http://dx.doi.org/10.7554/eLife.18767.004

	F80I (PDB 5KBN)	F83I (PDB 5KOM)
	P4_1_	P4_1_
cell dimensions		
α, β, γ (°)		
Resolution (Å)	37.9–2.48 (2.58–2.48)	38.0–2.69 (2.82–2.69)
R_merge_	0.085 (1.42)	0.083 (1.04)
I/σ	20.4 (2.0)	15.9 (2.0)
CC_1/2_	1.00 (0.851)	1.00 (0.819)
Completeness	0.999 (1.00)	0.999 (1.00)
Multiplicity	15.0 (15.2)	7.4 (7.5)
**Refinement Statistics**		
Resolution (Å)	37.9–2.48	38.0–2.69
No. Reflections	36356	28695
R_work_/R_free_	0.219 / 0.239	0.217 / 0.230
Ramachandran Favored	0.972	0.982
Ramachandran Outliers	0	0
RMS deviations		
Bond Lengths (Å)	0.0080	0.0084
Bond Angles (°)	1.19	1.28

### Inactivation of individual pores

**Figure 2.**
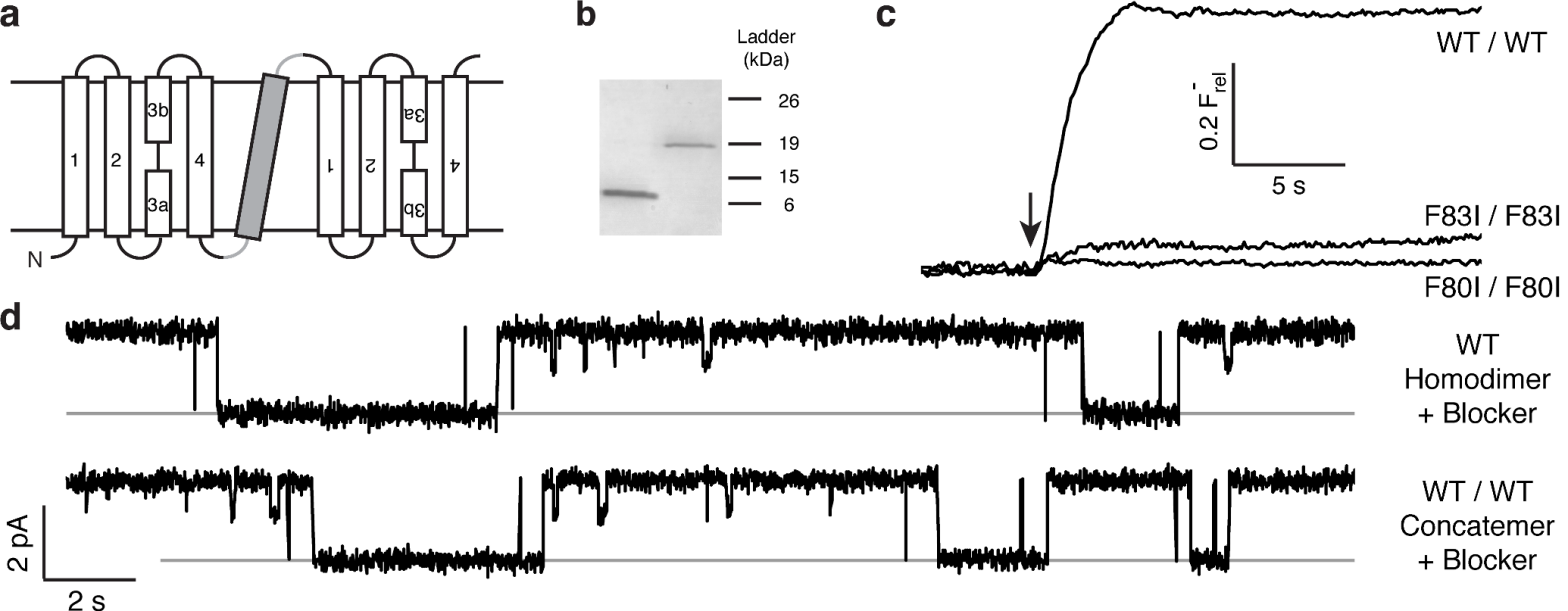
Proper assembly of fused-domain Ec2. (**a**) Cartoon of fused-domain construct, showing numbered transmembrane helices in Ec2 domains (open) fused by an 'inversion linker' containing a non-dimerizing glycophorin A helix (grey). (**b**) Coomassie-stained SDS PAGE gel of purified Ec2 homodimer (left lane) and concatemer (right lane), with soluble-marker ladder positions indicated. (**c**) Representative F^−^ efflux traces of indicated concatemeric constructs. (**d**) Representative single-channel recordings in the presence of 75–150 nM monobody, which induces long-lived nonconducting 'blocked' intervals; conducting intervals in these traces represent times when channels are free of monobody, with intrinsic open probability >95% (Stockbridge et al., 2014). Grey lines represent blocked current level. **DOI:** http://dx.doi.org/10.7554/eLife.18767.005

The results above point to the importance of the Phe-box side chains in F^−^ binding and permeation, and the physical organization of their associated F^−^ ions makes single-pore architecture implausible. But the homodimeric nature of Ec2 limits our ability to examine individual pores functionally, since any such mutation appears twice in the channel. We therefore constructed a tandem dimer by connecting two copies of the WT Ec2 sequence with a linker containing a foreign transmembrane helix to force an antiparallel arrangement of the two domains (Figure 2a, Figure 2—figure supplement 1). This construct, denoted 'WT/WT', mimics eukaryotic Fluc genes (Smith et al., 2015; Stockbridge et al., 2013), and it reprises an engineered homologue previously used to infer dual-topology assembly of Fluc channels (Stockbridge et al., 2013). The concatemer expresses poorly (~40 μg/L culture), but well enough for purification and reconstitution in liposomes and planar lipid bilayers (Figure 2b–d). The concatemer is functionally indistinguishable from the homodimeric WT channel with respect to unitary current, high intrinsic open probability, brief, infrequent closings, F^−^ selectivity, and submicromolar affinity block by monobody proteins (Figure 2—figure supplements 1–3). Moreover, the F80I / F80I and F83I / F83I double mutants with identical Phe substitutions in both domains - mimics of the mutant homodimers above - are likewise inactive (Figure 2c). Concatemeric Ec2 thus faithfully recapitulates the behavior of the corresponding homodimers.

**Figure 2—figure supplement 1.**
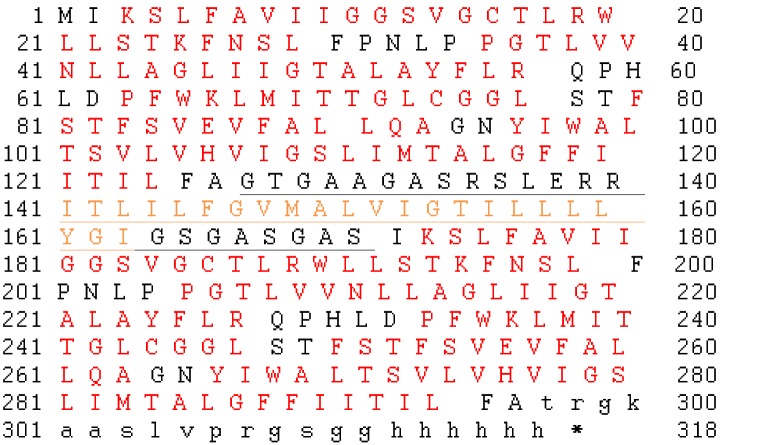
Amino acid sequence of WT/WT Ec2 concatemer. Fluc helices are indicated by red residues, and underlined residues indicate the artificial inversion linker, which includes a nondimerizing glycophorin A helix (orange residues). Lower case characters indicate the C-terminal His_6_ affinity tag and linker. **DOI:** http://dx.doi.org/10.7554/eLife.18767.006

**Figure 2—figure supplement 2.**
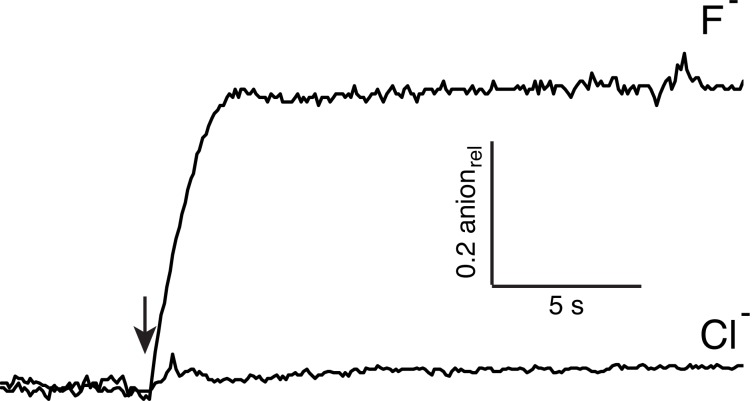
Concatemeric Ec2 retains F^−^ selectivity. Anion efflux traces shown for WT/WT concatemer, using liposomes loaded with 150 mM KF + 150 mM KCl. Efflux of the different ions was followed by F^−^- or Cl^-^-selective electrodes. **DOI:** http://dx.doi.org/10.7554/eLife.18767.007

**Figure 2—figure supplement 3.**
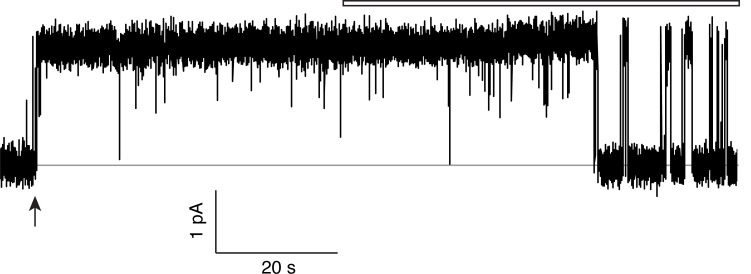
Monobody block to baseline. Single-channel traces of the WT/WT Ec2 concatemer here are used to demonstrate that monobody fully blocks the concatemer. Planar bilayers were formed and voltage was set to 200 mV. Recordings were begun in the absence of monobody to catch the first channel insertion into the bilayer (arrow). Initial monobody-free measurements show the high open probability of the channel. At the start of the open bar, monobody (200 nM) was added to the solution without stirring, and discrete monobody blocks commenced after the reagent diffused into solution. The level of current before channel insertion is indistinguishable from the level of monobody block, observed over at least four such experiments. **DOI:** http://dx.doi.org/10.7554/eLife.18767.008

This construct allows us to make channel-disruptive mutations along individual pores, and to assess whether channel function is retained by the unperturbed pore. The complete inhibition of F^−^ flux by either Phe mutant in the homodimer suggests a simple experimental design to test whether Fluc contains two functional pores. We use a liposome-based F^−^ efflux assay under 'Poisson-dilution' conditions (Stockbridge et al., 2013; Maduke et al., 1999; Wu et al., 2007), where most reconstituted liposomes carry only a single Fluc concatemer. Since efflux through a single channel is much faster than the time response of the F^−^ detection system, the assay provides a straightforward all-or-none indicator for the presence of functional channels; liposomes containing active channels release F^−^ immediately upon initiating efflux, regardless of whether one or two pores are active, while liposomes devoid of active channels, as with the functionally disabled Phe mutants (Figures 1d, 2c), show no F^−^ efflux.

**Figure 3.**
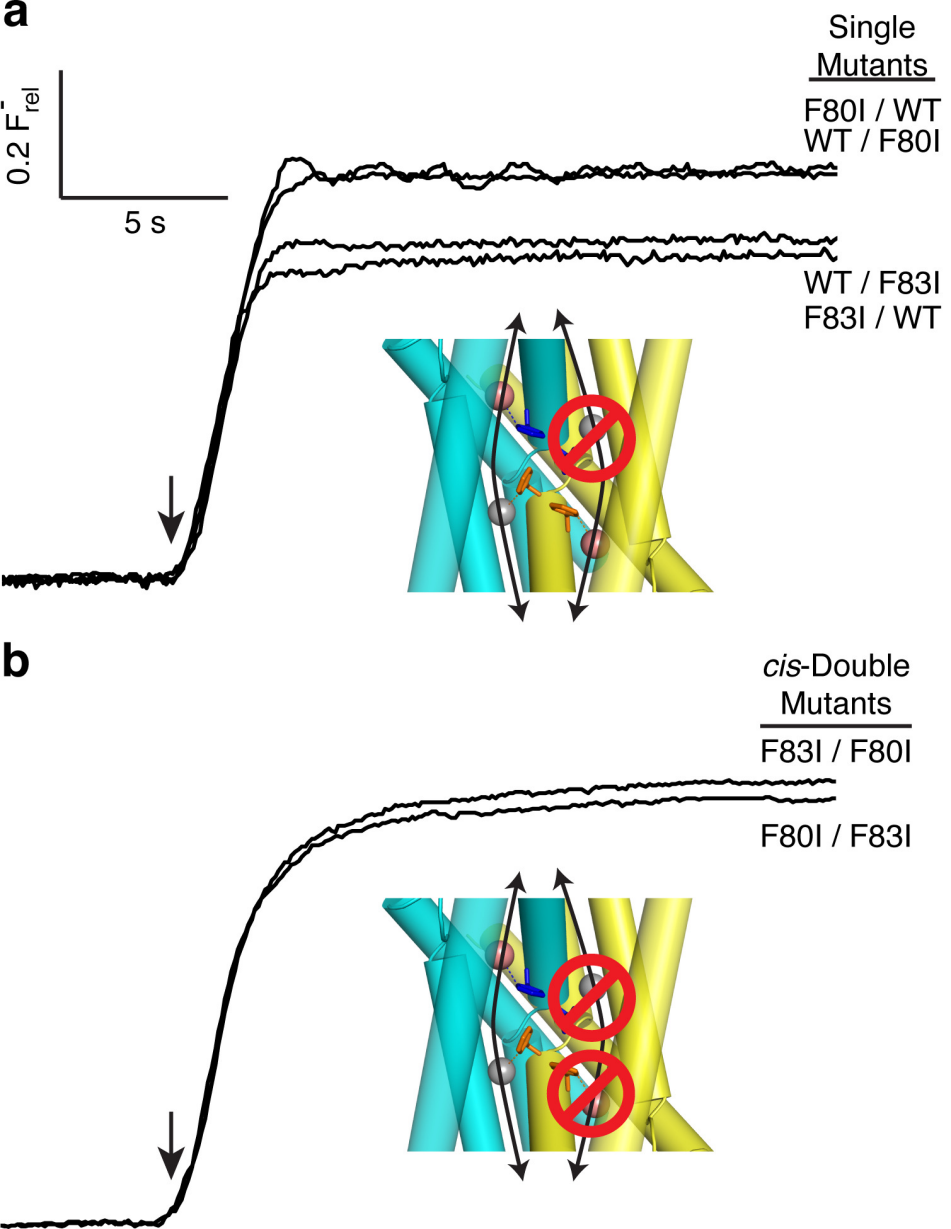
All-or-none assay for active F^−^ channels in concatemers. F^−^ flux traces are shown for concatemeric constructs containing single (**a**) or double (**b**) mutants. **DOI:** http://dx.doi.org/10.7554/eLife.18767.009

The flux behavior of the Phe-box mutants in the fused-domain channels meet all expectations of two-pore assembly, as Figure 3 illustrates. All four single Phe mutants, each of which leaves one pore unaltered, score active in these efflux experiments (Figure 3a). Since the two Phe residues from the same domain contribute to different pores, and thus each pore harbors Phe80 from one domain and Phe83 from the other, we can distinguish two classes of double mutants: *trans*, wherein the two mutations reside in different pores, or *cis*, with both mutations in the same pore. Two *trans* double mutants, F80I / F80I and F83I / F83I, are completely inactive, as shown above, while both *cis* double mutants (F80I / F83I, F83I / F80I) retain channel activity. (We were unable to test *trans* double mutants in which both Phe residues in the same domain were mutated, as these were biochemically intractable.) These results show that channels with both pathways mutated are inactive irrespective of the particular combination of mutations, while those mutated in only one pathway remain active, as expected for two-pore construction.

### Single-channel behavior of single-pore mutants

**Figure 4.**
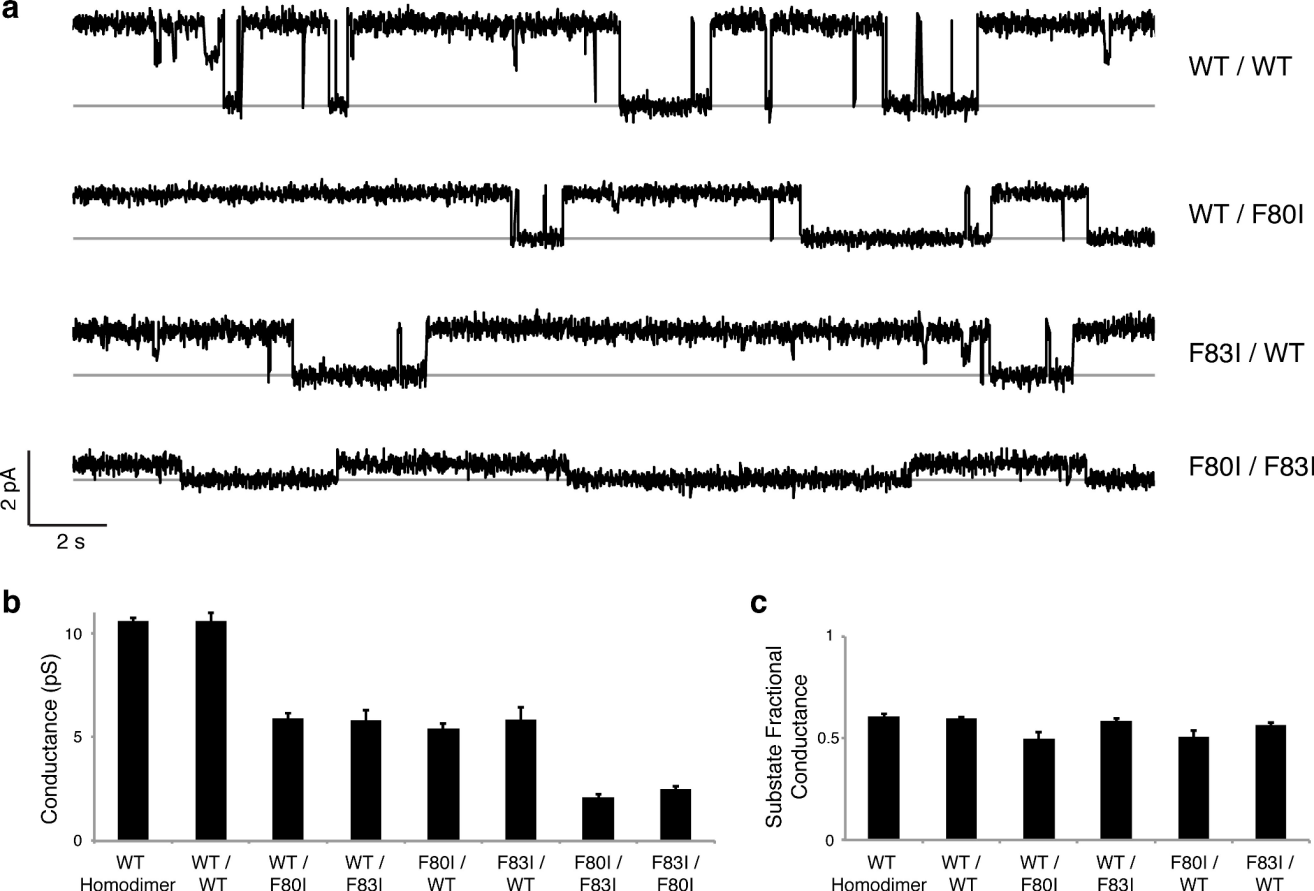
Single channels for single-pore mutants. (**a**) Illustrative single-channel traces in the presence of monobody blocker for indicated concatemers with two WT pores, and with three classes of single-pore mutants. Grey lines mark blocked state. (**b**) Summary of single-channel conductances of all channels scoring active in (Figure 3 ). (**c**) Summary for indicated constructs of substate conductance normalized to the full conductance level of the individual constructs. *cis*-Double-mutant channels were too small for substate analysis. Bars represent mean + s.e. of 10–14 separate channels. **DOI:** http://dx.doi.org/10.7554/eLife.18767.010

Although F^−^-efflux experiments clearly distinguish active from inactive channel mutants, their limited time-resolution (~1 s) precludes quantitative examination of F^−^ permeation rates. Single-channel electrical recording in planar lipid bilayers provides such information by directly comparing F^−^ current through WT channels and functionally active mutants. The results (Figure 4, Figure 4—figure supplement 1) are unambiguous: all single Phe-box mutants show unitary conductance of 5.7 + 0.3 pS, roughly half that of WT/WT fused-domain or WT homodimeric channels (10.6 + 0.1 pS). Moreover, the single-pore channels all recapitulate the high open probability, and monobody block of WT. We additionally note that the *cis* double mutants also show clear single-channel conductance that is nonetheless lower (2.3 + 0.2 pS) than the single mutants, possibly due to subtle, non-local structural disturbance of the conducting pore arising from the double mutation in the inactive pore. This ~2.5-fold drop in conductance, however, pales in comparison to the >10^4^-fold drop observed when adding a second mutation in the *trans* configuration (Figure 3).

**Figure 4—figure supplement 1.**
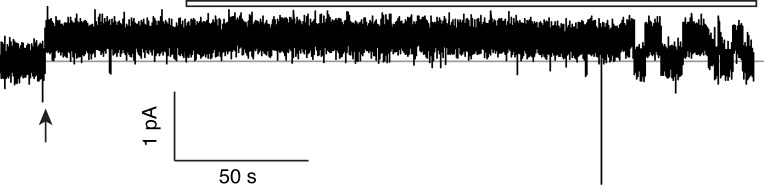
Monobody block to baseline. Single channel trace of F80I/F83I concatemer initially in the absence and later in the presence (open bar) of 200 nM monobody, as in Figure 2—figure supplement 3. The double phenylalanine single-pore mutant recaptures the WT/WT double-pore protein’s high open probability in the absence of monobody, and complete block by monobody. **DOI:** http://dx.doi.org/10.7554/eLife.18767.011

## Discussion

By examining the behavior of the Fluc Ec2 channel at a mechanistic level, these experiments now firmly establish the two-pore architecture suggested by crystal structures. We consider this to be a property of the entire Fluc family, a conclusion further supported by indications of genetic drift in non-symmetric Flucs and the asymmetrical effects of mutations in yeast Fluc channels (Smith et al., 2015; Stockbridge et al., 2015). The results also highlight the importance of the conserved Phe-box residues in crafting coordination spheres for which F^−^ ions are willing to shed their extensive aqueous hydration shells and enter the water-depleted confines of the ion-conduction pathway.

Two unexpected features of the mutant channels invite further comment. First, WT Ec2 channels show infrequent, brief excursions to a substate of ~6 pS, approximately one-half the conductance of the open channel (Figure 2d). It is natural to envision these as full closings of a single pore in a two-pore complex (Stockbridge et al., 2013), a picture demanding that such partial closings be absent in the single-pore mutants. However, this idea is plainly contradicted by the kinetically similar substates of ~3 pS appearing in the single-pore mutants here (Figure 4a,c). Instead, the substates in both wildtype and mutant channels must reflect a rare conformation that acts simultaneously on both pores to reduce F^−^ current in each, perhaps occurring in the wide, water-filled vestibules on the two ends of the channel complex. Second, the striking and aesthetically appealing edge-to-face arrangement of the Phe-box in the WT channel had originally suggested to us that these four sidechains mutually stabilize and orient each other so as to properly coordinate the F^−^ ions (Stockbridge et al., 2015). However, the Phe-mutant crystal structures here, while validating F^−^ coordination by the aromatic rings, refute the idea of a mutually stabilizing quartet, since the two Phe side chains remaining in each Ile substitution, though distant from each other, adopt identical orientations and crystallographic order as in the full Phe-box of WT (side chain rmsd 0.2 Å upon backbone alignment).

**Figure 5.**
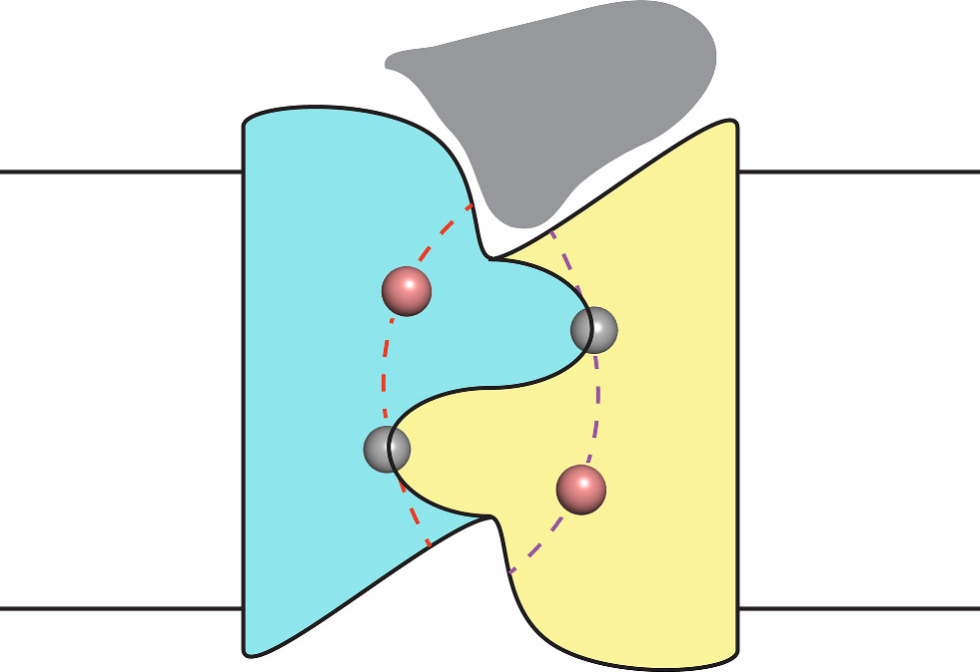
Proposal for trajectory of F^−^ permeation pathways. Cartoon envisions the dual-topology homodimeric channel with F^−^ ions (spheres) in the selective pores (dashed curves) connecting the vestibules. Monobody is shown (grey) bound in one of the vestibules, as in blocking experiments here, such that it occludes both pores simultaneously. **DOI:** http://dx.doi.org/10.7554/eLife.18767.012

Finally, despite the well-defined locations of the F^−^ ions and the span of the polar track residues, we acknowledge that the detailed trajectories of the two pores remain uncertain due to their very narrow bore. However, as indicated in the conjectural cartoon of Figure 5, it is likely that the pores originate and end in the two aqueous vestibules that give the channel a symmetrical hourglass shape. This suggestion follows from monobody interaction with Fluc channels. In crystal structures of both Fluc homologues, monobodies cap and intrude into the vestibules with long loops bearing their diversified sequences (Stockbridge et al., 2015); in channel recordings here, the S9 monobody inhibits both double- and single-pore channels fully to the zero-current level, as though both pores are occluded simultaneously by the blocker. This proposal thus envisions the vestibules as common regions of low selectivity from which F^−^ ions gain access to the separate F^−^-specific pathways.

## Materials and methods

### Biochemical

Fluc and Ec2 monobody S9 were expressed as previously described (Stockbridge et al., 2014; Stockbridge et al., 2015). For functional studies, purified Fluc was run over a S200 size-exclusion column equilibrated with 25 mM 4-(2-hydroxyethyl)-1-piperazineethanesulfonic acid (HEPES), 100 mM NaCl, 5 mM *n*-decyl-β-D-maltoside (DM), pH 7.0. Fluc channels were reconstituted into E. coli polar extract liposomes, and detergent was removed by dialysis against 25 mM HEPES, 300 mM KF, pH 7.0. For Cl^-^ efflux assays, these liposomes were then diluted 1:1 with 25 mM HEPES, 300 mM KCl, pH 7.0, sonicated to clarity, and then freeze-thawed 3 times. For crystallography, Fluc and monobody S9 were purified in 100 mM NaF, 10 mM HEPES, pH 7.0, with the Fluc solution also containing 5 mM DM. All Ec2 variants were constructed with standard PCR techniques.

### Fluc concatemer design

The base Ec2 construct used here, denoted 'WT', contains a single mutation (R25K) that increases expression but does not affect functionality (Stockbridge et al., 2015). The WT/WT Fluc concatemer was synthetically constructed with a hexahistidine tag, and designed so that the two Fluc domains would differ in nucleotide sequence while maintaining identical protein sequence. A transmembrane inversion linker based on a non-dimerizing glycophorin A variant (Lemmon et al., 1992) was used to join the two flux domains, as described for a Fluc heterodimeric homologue (Stockbridge et al., 2013).

### Anion efflux assays

F^−^ and Cl^−^ efflux assays were performed to detect anions released from pre-loaded proteoliposomes, as described (Stockbridge et al., 2015). Liposomes were prepared for Poisson-dilution conditions at low protein density (0.2 µg protein/mg lipid), such that on average only a single channel protein per proteoliposome is incorporated (Stockbridge et al., 2013), and ~50% of the liposomes are protein-free. The liposomes, loaded with 300 mM F^−^ or 150 mM F^−^ + 150 mM Cl^-^ were extruded 21 times through a 0.4 µm membrane, exchanged into 25 mM HEPES, 300 mM K-isethionate, pH 7.0 and diluted 20-fold into a flux buffer of the same composition also containing 1 mM of the appropriate halide. Efflux was initiated by adding 1 μM valinomycin and was followed electrochemically by monitoring anion appearance in the suspension using a F^−^ or Cl^−^ electrode. All traces are shown as relative anion efflux, calculated as the voltage output from the electrode normalized to the efflux signal upon dissolution of the liposomes with 30 mM octylglucoside. All efflux experiments were repeated 2–4 times, and representative traces are shown in figures.

### Single-channel recording

Single-channel recording was performed via a Nanion Orbit-mini planar bilayer system, using 70% 1-palmitoyl-2-oleoyl-phosphatidylethanolamine / 30% 1-palmitoyl-2-oleoyl-phosphatidylglycerol (Avanti Polar Lipids), 5 mg/mL in n-nonane to form bilayers. Single channels were inserted by addition of Ec2-reconstituted liposomes to the '*cis*' side of the bilayer, and current was recorded at +200 mV holding potential (*cis* side defined as zero voltage) in symmetrical solutions containing 300 mM NaF, 10 mM NaCl, 15 mM MOPS-NaOH pH 7.0, with 75–150 nM of monobody S9 added to the *cis* chamber unless otherwise noted. Recordings were low-pass filtered at 160 Hz, digitized at 1.25 kHz, and analyzed in Clampfit 10 after further digital filtering at 100 Hz. Channel conductance, averaged from 10–14 separate single-channel records under each condition, was measured from the difference between open vs blocked current in each recording. All recordings shown in figures are representative of behavior seen on many channels in multiple reconstitutions.

### Crystallography

Ec2 in complex with monobody S9 were crystallized as previously described (Stockbridge et al., 2015). Fluc dimers and S9 monobodies were individually purified and mixed at a 1:1.2 molar ratio at 10 mg/mL total protein. Crystallization well solutions were 100 mM *N*-(2-Acetamido)iminodiacetic acid, pH 6.2 (F80I) or 6.5 (F83I), 50 mM LiNO_3_, and 31% (F80I) or 36% (F83I) polyethylene glycol 600. Crystals were formed in hanging drops, mixing 1 μL each of protein and well solution, and incubating at 22°C for 14–21 days. Crystals were frozen in liquid nitrogen.

Datasets were collected at Advanced Light Source beamline 8.2.2. Diffraction images were processed in iMosflm (Battye et al., 2011), and data were merged and scaled using Aimless (Evans, 2011). The complete F80I dataset consisted of three different datasets from different positions along a single long crystal, merged in Aimless. Initial structure solution of both Phe mutants was done via molecular replacement using Phaser (McCoy et al., 2007) with PDB #5A43 as search model. Refinement was done with Refmac5 (Winn et al., 2003) and Phenix (Adams et al., 2010), using TLS refinement and local NCS averaging. Re-refinement of the previously collated WT Ec2 structure (Stockbridge et al., 2015) additionally used SAD data directly for additional phase information. Real-space refinement was done in COOT (Emsley et al., 2010), and Molprobity (Chen et al., 2010) was used to carry out model validation. Unbiased maps shown in Figure 1 are mFo-DFc maps output by Refmac5 using a model that had never had any fluorides introduced at any point in the refinement. Datasets from second crystals for both F80I and F83I that diffracted to similar resolution reproduce all the major structural findings here, particularly with regard to the pattern of bound F^−^ and the lack of perturbation of the overall Fluc structure.
